# Increases in alcohol consumption in women and elderly groups: evidence from an epidemiological study

**DOI:** 10.1186/1471-2458-13-207

**Published:** 2013-03-08

**Authors:** Lot M Geels, Jacqueline M Vink, Jenny HDA van Beek, Meike Bartels, Gonneke Willemsen, Dorret I Boomsma

**Affiliations:** 1 Department of Biological Psychology, VU University Amsterdam, Amsterdam, The Netherlands; 2 Neuroscience Campus Amsterdam, Amsterdam, The Netherlands; 3 EMGO+ Institute for Health and Care Research, VU University Medical Center, Amsterdam, Amsterdam, The Netherlands

**Keywords:** Alcohol consumption, Young adults, Women, Older adults, Epidemiology, Demographic variables, Lifestyle variables

## Abstract

**Background:**

In most Western countries, alcohol consumption continues to increase, specifically among women and older adults. Insight into these trends may aid intervention strategies. Here we present data on alcohol consumption by age and sex as well as associations between alcohol use and demographic lifestyle/traits. The data are from a large (N>16,000) population-based Dutch sample, ascertained based on the presence of twins in the family.

**Methods:**

A set of 16 indicators of normative and problematic alcohol use was assessed in participants of the Netherlands Twin Register between 2009–2012 (ages 18–97; 6,052 men; 10,535 women). Alcohol consumption and demographic/lifestyle traits, including educational attainment, work-related/financial stress, urbanization, religiousness, smoking/cannabis initiation, and BMI were described by age and sex. Associations were examined by regressing aspects of alcohol use on age, sex, their interaction, and demographic/lifestyle variables.

**Results:**

Age, sex, and initiation of cigarette and cannabis use were the most important predictors of alcohol use. Frequency of alcohol use was lowest between 18–25 years, with 3.2% of men and .6% of women drinking 6–7 times/week, and highest above age 65 years, with 30.6-32.7% of men and 20.2-22.0% of women drinking 6–7 times/week. Women consumed the lowest quantities of alcohol between 25–45 years, with a 5.7-5.9% prevalence of excessive drinking (>14 glasses/week), and the largest quantities between 55–65 years (15.5% excessive drinkers). Age at alcohol initiation, onset of regular drinking, and first alcohol intoxication were lowest between ages 18–25 years and highest above age 65 years. Among older participants, men initiated alcohol use and regular drinking earlier, and had lower age at first intoxication than women, but among young adults, no sex differences were observed.

**Conclusions:**

Alcohol consumption was high in the elderly Dutch population, especially among women. Alcohol initiation, onset of regular drinking, and first alcohol intoxication occur at increasingly younger ages, and the previous gap between men and women in age at alcohol initiation, onset of regular drinking, and first alcohol intoxication has closed almost entirely. Heavy alcohol use was most strongly predicted by older age, sex (male), and initiation of smoking and cannabis use.

## Background

Alcohol use is widespread in the Netherlands, with as much as 88% of the adult population having consumed alcohol in the past year [1]. The drinking pattern of the Dutch population is characterized by frequent but moderate alcohol consumption [2]. The Dutch drink on average 10 liters of alcohol per person each year, which is slightly higher than the average in the USA (9.4 liters) and below that observed across Europe (11 liters) [3,4]. The proportion of recent drinkers (in the last month) is higher in the Netherlands (76%) than across European countries (67%) and the USA (52%) [1,5,6]. Despite the general pattern of moderate drinking, the prevalence of heavy episodic drinking is 8.4% in Dutch adults (16% in men, 5% in women), which is somewhat higher than in the UK, the USA, and several European countries [4,7]. The proportion of problem drinkers is 9.4%, and the prevalence of alcohol use disorders is 5% in men and 1% in women, comparable with other western European countries [8,9]. Alcohol use contributes 4.5% to the total burden of disease in the Netherlands [10], as it increases risk of alcohol abuse and dependency and, when used excessively, contributes to numerous types of disease such as cancer, cardiovascular disease, and liver cirrhosis, as well as to alcohol-related injuries, e.g. traffic accidents [11].

This study presents a detailed assessment of alcohol use in the Dutch adult population, based on data collected between 2009–2011. Trends in alcohol use are examined and risk factors for increased alcohol use are identified based on associations between alcohol use indicators and demographic/lifestyle variables.

Alcohol use typically starts in adolescence, and drinking patterns vary across the lifespan. Before age 16, 72-85% of Dutch adolescents have initiated alcohol use [12,13]. The prevalence of heavy drinking (> 5 glasses on at least one occasion weekly) is highest between ages 18–24 [13], and young adults in this age group are at additional risk for alcohol abuse and alcohol-related problems in the college environment [14]. Overall, the prevalence of binge drinking (> 5 glasses per occasion) decreases between ages 15 and 64 years [13]. Above age 65, the number of drinkers declines [15]. Across all age groups, the prevalence of alcohol use is higher among men than women, and men have higher incidence of recent drinking and binge drinking [13]. Despite the relatively low number of drinkers above age 65 years, alcohol use disorders in older adults, especially women, seem to be a growing problem in the Netherlands [16]. The gap between men and women is similarly narrowing among young adults; between 2008–2009, the proportion of male heavy drinkers between ages 18–24 decreased substantially (from 37-30%), while remaining stable among women at 12% [13].

This age pattern is generally consistent with that observed in several important American studies on the epidemiology of alcohol use. In the USA, the prevalence of alcohol use is highest between age 25–44 and lowest in older age groups [17,18]. The college environment similarly puts young adults at additional risk for alcohol abuse and alcohol-related problems [19,20] and among older adults, about 1-3% are affected by alcohol use disorders [21,22].

As well as age and sex, socioeconomic circumstances and lifestyle factors are related to specific patterns of alcohol use. High educational attainment is associated with higher prevalence of alcohol use, but with lower levels of heavy alcohol use [23]. High levels of work-related stress have been related to increased quantity of alcohol consumed [24,25]. Financial stress may also be related to alcohol use, as observed in an elderly American sample [26], and in the Netherlands, alcohol dependence is more prevalent among the unemployed and those incapable to work [27]. Alcohol use and binge drinking occur more frequently in rural than in urban areas [13,28], in contrast with the globally observed association of higher alcohol use in urban areas [29]. Religiousness and higher frequency of church attendance are related to lower prevalence of heavy drinking [30-32]. Smoking cigarettes and cannabis use often co-occur with alcohol use [10,13,33]. The Netherlands have a unique ‘policy of tolerance’ towards cannabis, meaning that the substance itself is illegal, but possession of limited amounts of cannabis and selling by licensed establishments is not prosecuted [34].

Physical traits can also predict alcohol use. Body mass index (BMI) may be related to alcohol use through more than one route: in several European countries and the USA, low BMI is related to frequent consumption of small quantities of alcohol and to the preference of wine over beer or strong liquor, whereas high BMI is related to infrequently drinking large quantities of alcohol and preferring strong liquor [35,36].

Detailed insight in the latest trends in alcohol use is valuable to identify groups who are at relatively high risk for alcohol use disorders and to inform intervention strategies for such groups. Specifically, in this study we examine 1) previously observed age and sex effects on alcohol consumption in a large, recently-assessed sample; 2) interaction effects of age and sex on alcohol use; and 3) risk/protective factors for increased alcohol consumption, including demographic/lifestyle traits. These effects are assessed for a large set of normative as well as problematic alcohol variables.

Data were collected between 2009–11 in a large, population-based adult sample of twins (50%) and non-twins from the Netherlands Twin Register (NTR; N=16,587) [37]. Various aspects of alcohol use were examined as a function of sex and age-group, namely initiation and frequency of alcohol use, quantity of alcohol consumed, age at initiation and onset of regular drinking, preferred beverage, and situation-specific urges to drink. More severe aspects of alcohol use were also considered, including: number of alcohol intoxications, age at first alcohol intoxication, lifetime alcohol abuse disorder (AAD) symptoms, and hazardous drinking. Associations between alcohol use and age/sex were assessed by regression analysis, with demographic and lifestyle traits: educational attainment, work-related stress, financial stress, degree of urbanization, religiousness, smoking initiation, cannabis initiation, and body mass index (BMI) as covariates.

## Methods

### Sample

Participants were registered with the Netherlands Twin Register (NTR). Adolescent and young adult twins and their parents were initially recruited into the NTR through city councils in the Netherlands [38]. Additional recruitment efforts included the NTR website and yearly newsletter as well as publicity in the media [39]. Longitudinal survey collection in twins and their parents started in 1991. Participants were invited about every 3 years to complete a survey containing questions about health, lifestyle, personality and psychopathology [40]. The present study is based on data from the 8^th^ wave of survey collection that were collected between 2009 and 2011. After obtaining approval from the Medical Ethics Committee of the VU University Medical Center Amsterdam, all NTR participants aged 18 years and older, who were registered at a valid address, were invited to complete the survey (N=47,122). Participants first received a written invitation including a link to the webpage where they could log on to a web-based version of the survey with a unique, personal login name and password. If subjects did not access the web-based survey in the 6 weeks after the invitation, they received a paper version of the survey. Between 3–9 months after the paper versions of the survey were sent, subjects who had not responded received a reminder card by post, or a reminder by email (if an email address was available). Several groups of non-responders (e.g. twins from incomplete twin pairs) were reminded in a phone call. The survey was completed by 16,607 individuals (35% response rate). From these, 20 subjects were removed because they had only completed a small part of the survey. This resulted in a sample of 16,587 subjects from 7,308 families. The average age was 41.6 (SD=16.0; range 18–97), and 64% of the participants were women. Participants and their parents were mostly born in the Netherlands (92.7%). First and second generation immigrants from western countries other than the Netherlands formed 5.6% of the sample, and 1.7% were first or second generation immigrants from non-western countries.

Table 1 describes the sample. Twins (N=8,093), triplets (N=154), and 1 member of a quadruplet formed the largest group (N=8,248; 68.8-70.3% women), followed by parents (N=5,198; 59.2% women), non-twin siblings (N=1,898; 63.4% women), and spouses (N=875; 38.7% women) of multiples. Twins and higher-order multiples ranged in age between 18–97 years, parents between 30–94 years, non-twin siblings between 18–88 years, and spouses between 25–87 years.

**Table 1:** Age and sex of participants, stratified by family role

	***N***_***ind***_	***% women***	***Mean age (sd)***
Twins	8,093	68.8	33.5 (14.5)
Higher-order multiples (triplets/quadruplet)	155	70.3	25.9 (11.9)
Parents of twins	5,198	59.2	55.9 (8.1)
Spouses	875	38.7	44.3 (11.8)
Non-twin siblings	1,898	63.4	38.3 (13.9)
Other^1^	368	64.4	33.8 (10.1)
Total	16,587	63.5	41.6 (16.0)

^1^ includes multiples without information on co-multiples (N=35), participants registered without any family members (N=16), spouses of siblings of twins (N=28), children of twins/siblings (N=288), and a spouse of a child of a twin (N=1).

### Measures

#### Demographic and lifestyle variables

Educational attainment was available in 3 categories (primary school/lower vocational schooling’, intermediate vocational/upper secondary school’, and ‘upper vocational/university’; cf. Statistics Netherlands) [41] for 14,799 subjects. Student status at the time of data collection was based on a single item that asked if subjects were in college/university or high school (0 ‘no’, 1 ‘yes’). The frequency of work-related stress in the previous year was assessed (‘never’, ‘occasionally’, ‘regularly’, ‘constantly’, ‘not applicable’), as well as the degree of financial stress (‘little/none’, ‘moderate’, ‘severe’). Respondents who answered ‘not applicable’ to the question about work-related stress were included in the category ‘never’ (85% of these respondents did not have a job).

Degree of urbanization was based on address density in the residential area and measured on a scale of 1 to 5 (very high, high, moderate, low, very low) [42]. Following Statistics Netherlands, degree of urbanization was summarized in two categories (very low - moderate, and heavy - very heavy) [43]. Participants were asked if they were religious, to which they could answer ‘no’, ‘yes, not an active member of church/religious society’, or ‘yes, active member of church/religious society’.

Subjects were asked if they had ever smoked (‘no’, ‘yes, a few times to try’, and ‘yes’). The latter two categories were collapsed, creating a binary variable that indexed smoking initiation. Participants were also asked if they had ever used cannabis (0 ‘no’, 1 ‘yes’). BMI was calculated as weight_kg_ /height_m_^2^ and categorized into ‘underweight’ (BMI <18.5), ‘normal weight’ (BMI ≥18.5 & <25), and ‘overweight’ (BMI ≥25) [44]. BMI was not computed for women who were pregnant when they completed the survey (N=197).

#### Alcohol use

Participants were asked if they had ever used alcohol (‘no’, ‘a few times to try’, and ‘yes’). If subjects stated having ever used alcohol, they were asked at what age they had their first drink and at what age they had started drinking regularly (if at all). Subjects were asked how often they had used alcohol in the previous year (‘no’, ‘monthly or less’, ‘two to four times a month’, ‘two or three times a week’, ‘four or five times a week’, and ‘six times a week or daily’). Quantity of alcohol consumed in the previous year was measured as the number of glasses of beer, wine, and strong liquor, consumed on each day of a normal week. The reported number of glasses was summed into a continuous score for quantity of alcohol consumed per week. This variable was highly positively skewed and therefore categorized into ‘3 glasses or less’, ‘4-7 glasses’, ‘8-14 glasses’, ‘15-21 glasses’, and ‘more than 21 glasses’. The categories were based on the maximum weekly number of glasses recommended by the Health Council of the Netherlands (7 standard glasses for women, 14 for men), and criteria for excessive alcohol use (over 14 standard glasses per week for women, over 21 for men) [45,46].

Respondents were asked which alcoholic beverage they preferred (‘wine’, ‘beer’, ‘strong liquor’, ‘none’). Urges to drink alcohol in several situations (social situations, during/after dinner, after work, when relaxing, when concentrating, when under stress/pressure) were assessed, based on items about situation-specific urges to smoke [47]. Answer options for each situation were ‘not at all’, ‘mild’, and ‘strong’. If participants stated they had ever been intoxicated, they were asked at what age they had been intoxicated for the first time, and the number of times they had been intoxicated. Number of intoxications was severely skewed, and was therefore categorized into ‘once or twice’, ‘3-5 times’, ‘6-10 times’, ‘11-25 times’, and ‘>25 times’, based on the distribution in the sample. Lifetime prevalence of alcohol abuse and dependence (AAD) symptoms were assessed with the CAGE questionnaire, which consists of 4 items indexing AAD symptoms, that can be answered with ‘no*’,* ‘yes, not in the past year*’* or ‘yes, in the past year’ [48]. The last two categories were combined, resulting in four binary items for lifetime prevalence of each symptom. Item scores were summed and dichotomized into no (0) versus any (1–4) alcohol abuse and dependence symptoms [49]. Hazardous drinking was indexed by summing the 10 items of the Alcohol Use Disorders Identification Test (AUDIT), and dichotomizing at the cutoff score of 8, with scores above indicating hazardous drinking [50].

#### Analyses

The sample (N=16,587) was stratified by sex and age (age groups: 18–25, 25–35, 35–45, 45–55, 55–65, ≥65 years). In each sex by age group, the prevalence/distribution of demographic and lifestyle variables was computed. Subsequently, each case was assigned a proportional weight to correct for the most important deviations from the general population on demographic traits. Alcohol variables were described by sex and age while cases were weighted. For binary/categorical alcohol variables, prevalence/frequency distributions were computed using SPSS 18 [51]. For continuous alcohol variables, mean and standard deviation were computed in Mplus 5 while correcting for familial clustering, since standard deviations are underestimated if subjects are not independent [52,53]. Correlations among alcohol use indicators were also corrected for familial clustering in Mplus 5, as were regressions of alcohol use on demographic/lifestyle variables. Data on all predictor variables were available for 12,222 subjects. From this group, 75% (N=9,103) was randomly selected as the main dataset in which the regression analyses were carried out. The remainder of the sample (N=3,119) was used to cross-validate the results of the regression analyses in. These proportions were chosen to obtain the most reliable estimates of the regression coefficients in the main sample, while retaining a large enough validation sample. Age, sex (0 ‘male’, 1 ‘female’), an age*sex interaction term, and all other demographic/lifestyle variables were used to predict each aspect of alcohol use. The continuous alcohol measures (age at alcohol initiation, age at onset regular drinking, age at first alcohol intoxication) were predicted with linear regressions, and binary and ordered categorical alcohol measures (frequency of alcohol use, quantity of alcohol consumed, situation-specific urges to drink, number of alcohol intoxications, AAD symptoms, hazardous drinking) were predicted with logistic regressions. Alcohol initiation and preferred beverage were predicted with multinomial regressions, in which the reference categories were ‘no’ for alcohol initiation and ‘no preference’ for preferred beverage. The significance level was set at α=.01 in the main sample as well as in the validation sample, as a conservative criterion for replication of significant findings.

## Results

### Demographic and lifestyle variables

Table 2 shows prevalence/distribution of demographic and lifestyle factors, stratified by age-group and sex.

**Table 2:** Distribution or prevalence of demographic and lifestyle variables, stratified by age and sex

	***Age 18-25***		***Age 25-35***		***Age 35-45***		***Age 45-55***		***Age 55-65***		***Age 65 or older***	
	***Men N=1,235***	***Women N=2,499***	***Men N=839***	***Women N=1,769***	***Men N= 995***	***Women N= 1,891***	***Men N=1,192***	***Women N= 2,265***	***Men N= 1,255***	***Women N= 1,507***	***Men N= 536***	***Women N= 604***
*Educational attainment (N=14,799)*	%	%	%	%	%	%	%	%	%	%	%	%
Primary/lower vocat.	19.3	20.0	8.8	7.4	12.3	16.7	22.2	28.7	30.5	50.6	35.8	57.3
Intermediate vocat./ upper sec.	70.2	66.5	30.8	32.2	35.5	40.4	29.5	34.4	24.8	21.5	22.5	18.6
Upper vocat./university	10.5	13.5	60.4	60.4	52.2	42.9	48.3	36.8	44.7	27.9	41.6	24.1
*Work-related stress (N=13,956)*												
Never	61.0	53.8	49.0	43.0	51.0	46.7	48.6	48.6	58.2	60.9	71.1	78.3
Occasionally	32.7	36.8	35.6	36.8	33.1	38.0	38.8	36.1	30.9	27.7	22.1	15.8
Regularly	5.4	8.9	14.6	18.1	14.1	13.7	10.9	13.7	9.0	10.3	5.0	5.1
Constantly	.9	.5	.8	2.1	1.7	1.6	1.7	1.7	1.9	1.1	1.8	.8
*Financial stress (N=14,985)*												
None/little	73.8	64.6	71.7	65.5	71.7	66.8	71.2	67.2	78.5	77.4	84.6	84.4
Moderate	23.3	29.3	23.5	26.3	23.8	26.9	25.5	26.1	18.1	18.4	14.2	13.4
Severe	2.9	6.1	4.8	8.2	4.5	6.3	3.3	6.7	3.4	4.2	1.2	2.2
*Degree of urbanization (N=16,194)*												
Urban residential area	28.7	32.4	48.4	44.1	36.1	32.0	28.3	27.6	31.9	31.6	34.2	37.3
*Religiousness (N=16,180)*												
Not religious	62.0	59.8	61.9	57.2	55.9	47.6	47.3	39.2	41.2	31.9	31.9	21.2
Religious, not actively	26.7	28.9	26.2	29.1	29.6	37.4	35.1	41.7	40.1	44.9	39.1	37.8
Active church member	11.3	11.3	12.0	13.7	14.4	15.1	17.7	19.1	18.7	23.2	29.0	40.9
*Smoking initiation (N=15,527)*	52.6	47.0	62.1	55.9	60.7	55.4	68.9	72.7	84.3	76.5	83.3	57.0
*Cannabis initiation (N=15,122)*	41.6	29.5	47.9	31.2	30.6	20.6	16.6	10.9	11.9	7.3	4.0	1.3
*BMI (N=15,889)*												
Underweight	7.7	8.8	2.2	4.6	.2	2.0	.3	1.1	.2	1.3	.2	1.1
Normal weight	81.6	79.1	65.1	71.4	51.2	63.5	41.8	55.3	37.4	49.5	44.9	48.5
Overweight	10.7	12.1	32.7	24.0	48.6	34.5	57.8	43.5	62.4	49.2	54.9	50.4

The proportion of participants between ages 25–65 with low education was about the same as in the general population (23% versus 27%, respectively), while high education was somewhat more prevalent than in the general population (45% versus 32%) [54]. Among participants between 18–25 years old, the majority (68%) were students and had not yet completed school or university. This percentage was higher than that observed in the general population (40%) [55]. Work-related stress (in the previous year) was most prevalent between ages 25–35 (51.0% in men; 57.0% in women) and least prevalent above age 65 years (28.9% in men, 21.7% in women). The majority of the participants had experienced little or no financial stress in the previous year (64.6-84.6%). The proportion of participants who lived in densely populated (urban) areas ranged between 27.6–48.4% and was somewhat lower than in the general population [43]. Between ages 18–25, about 40% of participants was religious (either non-actively; about 28%, or actively; about 11%). Religiousness was most prevalent above age 65 years (68.1% in men, 78.8% in women). Overall, religiousness was slightly less prevalent than in the general population (about 1-10% difference) [56].

Between ages 18–25, about half of the participants had initiated smoking cigarettes. Among men, this proportion was highest above age 65 (83.3%). Among women, the prevalence of smoking initiation was highest between ages 55–65 (76.5%), but lower above age 65 years (57.0%). Overall, the prevalence of smoking initiation was 63.2%, similar to the prevalence in the general population (60.0%) [57]. Cannabis initiation was most prevalent between ages 25–35 years (47.9% in men, 31.2% in women), and least prevalent above age 65 years (4.0% in men and 1.3% in women). The prevalence of cannabis initiation in women was highly similar to that in the general population, while in men, it was slightly lower (the largest difference was 9%) [58]. BMI was higher in the older than in the younger age groups. Above age 45 years, more than half of the men were overweight (54.9-62.4%). Women were mostly classified in the normal weight range, except above age 55 years, where about 50% of women were overweight. Overweight was slightly less prevalent than in the general population, mainly in the younger age groups [44].

### Alcohol use

Each case was assigned a proportional weight based on educational attainment, since the sample distribution of this variable deviated most from the distribution in the general population. Table 3 shows prevalence/distribution or mean and standard deviation of alcohol use indicators, stratified by age and sex (see Additional file 1: Table A1 for prevalence/means in the unweighted sample).

**Table 3:** Prevalence/distribution or mean and standard deviation of alcohol use indicators, by age and sex (subjects weighted for educational attainment)

		***Age 18-25***		***Age 25-35***		***Age 35-45***		***Age 45-55***		***Age 55-65***		***Age 65 or older***	
		***Men N=1,222***	***Women N=2,493***	***Men N=829***	***Women N=1,754***	***Men N= 978***	***Women N= 1,874***	***Men N=1,201***	***Women N= 2,294***	***Men N= 1,264***	***Women N= 1,523***	***Men N= 543***	***Women N= 612***
*Alcohol initiation (N=*16,243*)*		*%*	*%*	*%*	*%*	*%*	*%*	*%*	*%*	*%*	*%*	*%*	*%*
No		3.0	4.1	1.7	5.0	2.1	5.1	2.8	4.7	2.1	5.9	2.9	14.3
A few times to try		5.4	7.7	3.2	7.2	4.9	9.2	1.8	7.1	1.7	5.8	2.5	8.7
Yes		91.6	88.2	95.1	87.7	93.0	85.7	95.4	88.2	96.1	88.4	94.7	77.0
*Frequency of alcohol use (N=*15,972*)*													
Never		7.4	10.7	6.2	18.8	7.3	16.6	6.8	13.7	5.1	14.5	9.6	23.8
Monthly or less		13.0	28.1	14.7	33.7	16.6	28.4	9.4	19.5	9.2	16.3	9.8	15.0
2–4 times a month		34.9	41.8	34.3	26.4	26.6	24.4	21.2	20.1	14.5	16.0	16.1	12.4
2–3 times a week		34.1	17.1	31.9	15.4	27.7	18.0	29.3	21.6	24.3	18.7	16.3	18.0
4–5 times a week		7.5	1.8	8.2	3.7	10.8	6.9	14.2	10.8	16.2	12.5	15.5	10.7
6–7 times a week		3.2	.6	4.8	2.0	11.0	5.6	19.1	14.3	30.6	22.0	32.7	20.2
*Weekly alcohol quantity (N=*12,828*)*													
3 glasses or less		22.0	39.5	23.9	52.1	24.6	46.4	20.5	35.6	14.6	29.3	17.5	30.7
4–7 glasses		20.1	29.8	27.0	28.4	27.6	30.3	24.0	31.8	23.2	29.4	28.5	29.6
8–14 glasses		23.4	19.1	28.2	13.8	25.7	17.4	31.5	22.3	29.9	25.8	26.4	25.4
15–21 glasses		17.4	6.3	10.5	3.0	12.8	3.4	13.6	7.8	18.6	10.5	15.1	11.3
More than 21 glasses		17.2	5.4	10.3	2.7	9.3	2.5	10.4	2.7	13.8	5.0	12.5	3.1
*Preferred beverage (N=*14,407*)*													
Wine		2.3	37.3	14.0	56.5	22.0	66.3	31.3	79.9	37.9	82.4	46.3	85.3
Beer		70.4	13.7	64.0	11.4	53.3	8.5	49.4	5.4	41.4	4.0	25.8	2.3
Strong drinks		13.0	29.2	8.5	19.8	8.6	14.0	7.1	5.2	8.3	5.1	12.7	4.5
No preference		14.3	19.7	13.5	12.3	16.2	11.2	12.1	9.5	12.4	8.4	15.2	7.9
*Urges to drink alcohol*													
*Social situations (N=15,052)*	No	20.0	23.1	12.4	23.5	18.1	26.9	19.1	30.2	21.3	31.9	32.2	35.9
	Mild	52.5	58.3	55.2	56.4	56.0	57.4	62.6	58.0	66.0	59.5	60.4	58.3
	Strong	27.5	18.5	32.4	20.1	25.9	15.7	18.3	11.8	12.7	8.6	7.5	5.8
*At dinner (N=14,968)*	No	72.8	79.6	64.0	69.8	64.7	69.0	63.6	64.7	55.7	55.5	47.7	45.6
	Mild	26.5	19.2	33.2	26.8	31.6	27.1	32.2	31.0	39.3	40.2	46.2	51.0
	Strong	.7	1.2	2.8	3.4	3.7	3.9	4.3	4.2	5.0	4.3	6.1	3.3
*After work (N=14,828)*	No	66.1	90.5	75.4	90.5	79.7	91.2	81.3	87.7	73.9	81.3	78.0	82.2
	Mild	26.9	8.6	21.2	7.9	17.8	7.0	15.2	9.6	22.2	15.4	18.5	16.4
	Strong	7.0	.9	3.4	1.6	2.5	1.8	3.4	2.7	3.9	3.3	3.5	1.4
*Relaxing (N=15,000)*	No	36.9	53.3	39.6	54.2	39.6	51.9	28.7	41.7	25.8	40.5	36.8	38.1
	Mild	51.5	41.7	51.7	41.8	48.8	42.6	62.1	52.5	67.3	53.9	59.5	58.6
	Strong	11.7	5.0	8.7	4.1	11.6	5.5	9.1	5.8	6.8	5.6	3.7	3.3
*Concentrating (N=14,667)*	No	97.3	98.3	98.0	99.0	97.4	99.1	97.6	99.0	96.3	96.5	93.5	96.3
	Mild	2.6	1.6	1.7	.9	2.3	.8	2.3	1.0	3.4	3.3	6.3	3.7
	Strong	.1	.1	.3	.1	.3	.1	.1	.1	.3	.2	.2	0
*Under stress (N=14,705)*	No	79.8	84.0	80.2	84.6	82.2	82.9	78.1	78.2	74.4	72.9	75.1	73.4
	Mild	16.4	13.0	16.2	11.9	15.5	13.9	18.0	17.6	20.6	21.7	19.9	21.2
	Strong	3.8	3.0	3.6	3.5	2.3	3.2	3.9	4.2	5.0	5.4	5.0	5.4
*No. of intoxications (N=8,885)*													
Once or twice		29.3	39.6	14.7	32.1	18.6	37.9	22.3	44.5	25.6	50.9	36.3	55.3
3–5 times		26.4	32.2	21.0	30.9	21.7	34.3	32.8	35.8	33.5	29.7	31.8	28.5
6–10 times		18.3	13.7	23.2	17.1	19.9	16.2	23.9	13.1	22.0	12.1	18.0	10.6
11–25 times		13.6	7.8	14.2	11.4	16.0	7.1	10.5	3.6	10.5	4.4	9.0	4.9
More than 25 times		12.5	6.7	26.9	8.4	23.8	4.5	10.5	3.0	8.4	2.9	4.8	.8
*Alcohol abuse disorder symptoms (N=15,227)*		35.2	20.9	41.4	18.2	32.5	17.1	34.1	22.2	36.6	23.3	28.0	16.2
*Hazardous drinking (N=15,467)*		29.5	13.0	22.7	7.2	19.1	6.6	16.6	9.8	18.6	9.4	12.7	5.5
		*mean (sd)*	*mean (sd)*	*mean (sd)*	*mean (sd)*	*mean (sd)*	*mean (sd)*	*mean (sd)*	*mean (sd)*	*mean (sd)*	*mean (sd)*	*mean (sd)*	*mean (sd)*
*Age at alcohol initiation (N=*15,155*)*		14.5	14.6	15.1	15.5	15.5	16.5	15.4	16.5	16.3	17.8	18.1	20.4
		(2.0)	(1.7)	(2.4)	(2.4)	(2.5)	(2.9)	(2.3)	(3.0)	(2.8)	(4.4)	(4.2)	(6.8)
*Age at onset regular drinking (N=*8,483*)*		16.7	16.9	18.2	19.6	19.1	22.6	20.4	25.5	22.6	29.6	27.8	33.3
		(1.2)	(1.6)	(3.0)	(4.9)	(4.0)	(6.3)	(5.8)	(8.9)	(8.4)	(11.1)	(12.0)	(12.7)
*Age at first intoxication (N=10,102)*		16.5	16.6	17.4	18.9	18.2	20.9	18.7	22.2	20.4	25.3	24.3	32.2
		(1.3)	(1.5)	(2.7)	(3.4)	(3.0)	(5.4)	(3.9)	(7.0)	(5.0)	(8.3)	(8.3)	(11.0)

Around 94-98% of the participants had initiated alcohol use, except women aged 65 years or older (85.7%). Across all age groups, more men had initiated alcohol use than women. Frequency of alcohol use was lowest among young adults (age 18–25 years), with 3.2% of men and .6% of women drinking 6–7 times a week. Men above age 65 years drank most frequently (32.7% drank 6–7 times per week). The proportion of women who drank 6–7 times a week was highest above age 55 years (20.2-22.0%). Across all age groups, men drank more frequently than women. Quantity of alcohol showed somewhat different age patterns in men and women. In men, drinking more than the recommended 14 glasses per week occurred most often between ages 18–25 years (34.6%) and nearly as often between ages 55–65 years (32.4%). The proportion of men who drank more than 14 glasses a week was smallest between 25–35 years (20.8%). Excessive drinking (>21 glasses a week) occurred least often in men aged between 35–45 years (9.3%), and most often between ages 18–25 years (17.2%). Among women, quantity of alcohol consumed was lowest between ages 25–35 years, with 19.5% drinking more than the recommended 7 glasses per week, and 5.7% excessive drinkers (>14 glasses per week). Women drank the largest quantities above age 55 years, with about 40% drinking more than the recommended 7 glasses per week, and about 15% excessive drinkers (>14 glasses per week). Men drank larger quantities of alcohol than women across the entire age range.

Wine was more popular above age 65 than in the youngest age group (46.3 versus 2.3% in men, 85.3 versus 37.3% in women). Among men, beer was the most popular beverage, while women mostly preferred wine, followed by strong liquor. The urge to drink in social situations was reported most often (64.1 – 87.6%), and the least often reported urge to drink was while concentrating (.9 - 6.5%). The prevalence of urges to drink in different situations was slightly higher at older ages, especially among women. Generally, men experienced more urges to drink alcohol than women.

The number of alcohol intoxications was highest for individuals between 25–35 years; 26.9% of men and 8.4% of women had been intoxicated more than 25 times in this age group, compared to 4.8% of men and .8% of women above age 65 years. Men reported more alcohol intoxications than women. In men, lifetime AAD symptoms were most prevalent between ages 25–35 years (41.4%) and least prevalent above age 65 years (28.0%). Among women, lifetime AAD symptoms occurred most frequently between ages 55–65 years (23.3%), and least frequently above age 65 years (16.2%). Lifetime prevalence of AAD symptoms was higher in men than in women. Hazardous drinking occurred most frequently between ages 18–25 years (29.5% in men, 13.0% in women), and least often above age 65 years (12.7% in men, 5.5% in women). Hazardous drinking was more prevalent in men than in women.

Between ages 18–25 years, age at alcohol initiation was lowest, and highly similar for men and women (14.5 in men, 14.6 in women). Among individuals aged 35 years and older, men initiated alcohol use at a younger age than women. Average age at initiation was highest for individuals aged 65 years or older (18.1 years in men and 20.3 in women). Similarly, average age at onset of regular drinking was lowest in the youngest age group (16.7 years in men, 16.8 in women) and highest above age 65 years (28.2 years in men, 33.8 in women). Average age at first intoxication showed the same pattern: it was lowest between ages 18–25 years (16.5 in men, 16.6 in women) and highest above age 65 years (24.2 in men, 31.6 in women). With the exception of the 18–25 age group, men were younger than women at first alcohol intoxication.

**Figure 1:**
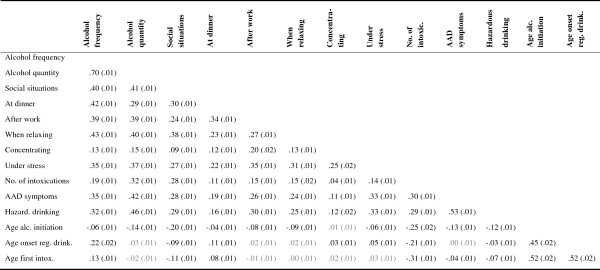
**Correlations between alcohol use indicators (N=16,267).***Note.* Correlations are standardized covariances. Standard errors are in parentheses. Black font: significant at α=.01; grey font: non-significant.

Figure 1 shows the correlation coefficients between all indicators of alcohol use, except the nominal alcohol variables (initiation of alcohol use and preferred beverage).

Correlations that were significant at α=.01 are shown in black font and non-significant correlations in grey font. Nearly all aspects of alcohol use were significantly inter-related. Age at onset of regular drinking and age at first alcohol intoxication were least strongly associated with other alcohol variables (absolute values of correlations ranged between .00 and .52). Frequency of alcohol use was most strongly related to other alcohol variables (absolute values of correlations ranged between .06 and .70).

### Associations of alcohol use with demographic and lifestyle variables

**Figure 2:**
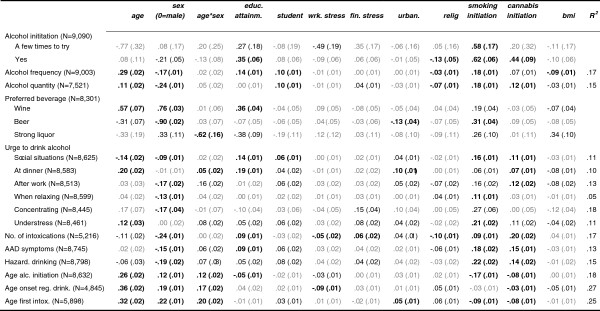
**Regression (β) of alcohol use on demographic and lifestyle variables.***Note.* β (beta): standardized regression coefficients, with standard errors in parentheses. Betas in bold font were significant at α=.01 in the main sample and in the validation sample. Betas in regular black font were significant (at α=.01) in the main sample but not in the validation sample. Betas in grey were not significant (at α=.01).

Associations (standardized regression coefficients; β) between each aspect of alcohol use and demographic and lifestyle variables are shown in Figure 2.

The regression (β) coefficients that were significant at α=.01 in both the main sample and the validation sample are shown in bold font. The regression coefficients that were significant in the main sample but not in the validation sample are shown in regular font, and the non-significant coefficients are shown in grey (see Additional file 2: Table A2 for the cross-validation results). The regression analyses confirmed that age at alcohol initiation, onset of regular drinking, and first alcohol intoxication were lower in younger, than in older participants (betas between .26 and .36). Older participants drank more frequently and larger quantities of alcohol use, and more often preferred wine (absolute beta values ranged between .11 and .57). Urges to drink alcohol at dinner and under stress were more prevalent among older participants (β=.20 and β=.12 respectively). In contrast, the urge to drink in social situations was more prevalent in younger participants (β=-.14). Preference for strong liquor decreased over age in women, but this association was not observed in men (β=-.62). The urge to drink at dinner was more prevalent in older than in younger women, but in men, no association with age was observed (β=.05). Men scored higher than women on nearly all aspects of alcohol use (betas between -.09 and -.24). Men were also younger at alcohol initiation, onset of regular use, and first intoxication (betas between .12 and .22). This sex difference was significantly smaller in the young, than in the older participants (betas ranged between .12 and .20). Women mostly preferred wine (β=.76), and among men, beer was the most popular beverage (β=-.90).

High educational attainment, being a student, experiencing financial stress, and high degree of urbanization were associated with higher levels of alcohol use (betas between .06 and .36), while work-related stress, religiousness, and high BMI were related to lower levels of alcohol use (betas between -.03 and -.13). Initiation of smoking and cannabis were strongly, positively related to many aspects of alcohol use (betas between .07-.62), as well as to early age at alcohol initiation and age at first intoxication (betas between -.03 and -.17).

The proportion of variance explained by the demographic and lifestyle variables ranged between 5% (urge to drink while relaxing) and 27% (age at onset regular drinking).

## Discussion

A wealth of information on drinking patterns in the Dutch population was obtained from the analysis of multiple alcohol consumption variables in a large, population-based sample of adults, ranging in age between 18 and 97 years. Frequency and quantity of alcohol use, urges to drink, and indicators of more severe alcohol use were strongly associated with each other, but less strongly with age at initiation of alcohol use, onset of regular drinking, and first alcohol intoxication. With respect to effects of sex and age, we highlight the most important findings.

Alcohol consumption was high in men and women above age 55, suggesting that the trend of increased alcohol consumption in older Dutch adults is continuing [16]. Specifically, men and women (above age 55) drank more frequently than young adults. Excessive drinking (>14 glasses of alcohol per week) was substantially more prevalent among women above age 55 than in the young adult age group. The prevalence of excessive drinking was lowest in women between ages 25–35 years, when they typically have children [59], and the high proportion of excessive drinking in elderly women may be explained by their having more opportunity to drink when their children enter adulthood. Increases in alcohol consumption in older adults may be due to the growing number of healthy life years, in combination with a higher average income, which has increased substantially over the past years in the elderly Dutch population [60,61]. Problematic alcohol use poses greater health risks for older adults than for younger individuals, since with increasing age, tolerance to the effects of alcohol declines. In addition, older adults may use medication that interacts negatively with alcohol, or suffer from chronic physical or psychological illness, such as diabetes or mood problems, that may be worsened by alcohol [22,62].

Recent data suggest that the gap between male and female drinking is narrowing among young adults [13,63,64]. This trend is corroborated by the finding that among participants between 18–25 years old, women initiated alcohol use, started drinking regularly, and reported first alcohol intoxication at the same ages as men. Declining sex differences are mainly caused by increases in alcohol use in women, which may result from women having more freedom and financial independence. Drinking among women has also become more socially accepted than several decades ago, and young women nowadays may have fewer family responsibilities [65]. This trend has important consequences for public health, since women are more susceptible to the harmful effects of alcohol, such as liver and heart disease, than men [66].

Additionally, since the 1940s-50s, when the individuals in the highest age category were adolescents, alcohol initiation, onset of regular drinking, and first intoxication have been occurring at increasingly younger ages. This is in line with the increase in alcohol consumption in the Dutch population observed between 1950 and 1980, specifically of beer and imported wine [67,68]. The increase was likely due to strong economic growth and increases in international commerce during this period [69]. It should be noted, however, that the differences in age at alcohol initiation between young and old participants may be slightly overestimated due to retrospective reporting bias [70,71].

Besides age and sex, initiation of cigarette and cannabis use were the most striking risk factors for increased alcohol use, early alcohol initiation and age at first alcohol intoxication. These associations are likely explained by an underlying genetic vulnerability for substance use [72]. Individuals who use multiple substances often meet more criteria for abuse or dependence than those who only use a single substance. Moreover, using more than one substance is associated with increased health risks, due to the combined harmful effects of alcohol and cigarettes or cannabis [73,74].

Some limitations of this study should be noted. Self-report data may be imprecise due to memory errors or underreporting of substance use [75]. Moreover, 50% of the sample were twins. Twins are born in all population groups, which makes having a twin in the family a suitable criterion for families to be included in a population-based sample [76]. Nevertheless, twin studies have been criticized since pregnancy and birth of twins carry increased risks and twins generally have a lower birth weight than singletons [77]. These are risk factors for behavioral problems, which are strongly related to alcohol use [78,79]. However, studies in several European countries have not observed differences in externalizing problems between twins and singletons during childhood and adolescence [80,81].

The participants in the 65+ age group ranged in age between 65 and 97 years. As age increases, so does the risk of cognitive impairment, which may have lead to imprecise reports of alcohol use [22]. Moreover, although the cutoff for hazardous drinking based on the AUDIT questionnaire was lowered for the older participants, it may not be appropriate for this entire age range, since with increasing age, the ability to metabolize alcohol decreases [22,62]. Similarly, the CAGE questionnaire was developed in a sample of males under age 61 [48], and may therefore not be entirely appropriate for the oldest participants.

Excessive alcohol use was based on number of glasses consumed per week, and thus may have been somewhat inaccurate, since the criteria for excessive alcohol use are based on standard glasses of alcohol [45], which were not specifically reported in the survey.

Because of the large number of predictor variables, no other interaction effects were examined than between age and sex, while these may be relevant in the prediction of alcohol consumption. For example, those above age 55 years who seek treatment for alcohol use disorders are more often highly educated than treatment seekers below age 55 years [16]. Similarly, alcohol dependence is positively related to high education in women, but not in men [27].

To summarize, we observed that elderly Dutch men and women continued to drink alcohol more frequently than young adults, and excessive drinking was substantially more prevalent in elderly women than in young adult women. Until now, alcohol prevention campaigns have predominantly targeted adolescents and young adults, but the high levels of alcohol consumption among older adults warrant prevention and intervention campaigns aimed specifically at this age group [82]. Initiation of alcohol use, onset of regular drinking, and first alcohol intoxication occur at increasingly younger ages, and the gap that previously existed between men and women in age at alcohol initiation, age at onset of regular drinking, and age at first alcohol intoxication continues to close.

## Conclusions

In the Netherlands, men and women above age 55 drink alcohol more frequently than young adults, and excessive drinking is substantially more prevalent in these women than in young adult women. Alcohol initiation, onset of regular drinking, and first alcohol intoxication occur at increasingly younger ages, and women continue to catch up to men with respect to when they initiate (regular) alcohol use and first get intoxicated. These trends have negative implications for public health, since women and older adults are particularly susceptible to the harmful effects of alcohol.

## Competing interests

The authors declare that they have no competing interests.

## Authors’ contributions

LG and JvB collected the data, under supervision of JV, GW, MB, and DB. DB initiated the study. LG conducted the analyses and drafted the manuscript. JvB, JV, GW, MB, and DB commented on the manuscript. All authors read and approved the final manuscript.

## Pre-publication history

The pre-publication history for this paper can be accessed here:

http://www.biomedcentral.com/1471-2458/13/207/prepub

## Supplementary Material

Additional file 1: Table A1 Prevalence/distribution or mean and standard deviation of alcohol use indicators, by age and sex, in unweighted subjects. Click here for file

Additional file 2: Table A2 Cross-validation of regression (β) of alcohol use on demographic and lifestyle variables. Click here for file
