# Supplementary material for: Increases in alcohol consumption in women and elderly groups: evidence from an epidemiological study
Source: BMC Public Health. 2013 Mar 8;13:207. doi: 10.1186/1471-2458-13-207 (PMC3720280; doi:10.1186/1471-2458-13-207)
Supplement: Additional file 1: Table A1 — Prevalence/distribution or mean and standard deviation of alcohol use indicators, by age and sex, in unweighted subjects. [file 1471-2458-13-207-S1.pdf]

Table A1 Prevalence/distribution or mean and standard deviation of alcohol use indicators, by age and sex, in unweighted subjects

		Age 18-25		*Age 25-35*		*Age 35-45*		*Age 45-55*		*Age 55-65*		*Age 65 or older*	
		Men N=1,235	*Women N=2,499*	*Men N=839*	*Women N=1,769*	*Men N= 995*	*Women N= 1,891*	*Men N=1,192*	*Women N= 2,265*	*Men N= 1,255*	*Women N= 1,507*	*Men N= 536*	*Women N= 604*
Alcohol initiation (N=16,239)		*%*	*%*	*%*	*%*	*%*	*%*	*%*	*%*	*%*	*%*	*%*	*%*
No		2.6	3.6	1.6	3.8	2.2	4.8	2.8	4.5	2.0	6.0	2.9	14.3
A few times to try		4.4	7.0	3.1	7.0	4.2	8.5	1.9	6.8	1.6	5.7	2.5	8.7
Yes		93.0	89.4	95.4	89.2	93.6	86.7	95.3	88.7	96.3	88.3	94.7	77.0
Frequency of alcohol use (N=15,321)													
Never		3.5	6.0	3.9	12.9	4.3	11.4	3.9	9.0	2.9	9.0	6.9	10.0
Monthly or less		12.2	28.0	13.2	33.4	15.9	29.2	9.2	19.5	8.7	17.0	10.1	17.7
2-4 times a month		36.1	42.9	35.2	29.3	27.7	26.2	21.4	20.8	14.8	17.2	16.6	14.6
2-3 times a week		36.2	20.1	34.2	17.5	29.8	19.5	29.8	23.3	24.5	19.9	16.8	21.2
4-5 times a week		7.8	2.4	8.4	4.6	11.3	7.8	15.6	11.7	17.1	13.5	16.0	12.6
6-7 times a week		4.2	.6	5.0	2.4	11.0	6.0	20.1	15.7	32.1	23.4	33.7	23.9
Weekly alcohol quantity (N=13,026)													
3 glasses or less		21.5	39.3	23.3	51.8	25.3	46.5	20.1	34.8	14.3	28.9	17.5	30.7
4-7 glasses		18.6	29.2	28.6	28.7	29.2	30.0	24.6	32.1	23.1	29.4	28.5	29.6
8-14 glasses		25.0	19.9	28.4	13.9	26.0	17.7	31.6	22.7	29.5	26.3	26.4	25.4
15-21 glasses		16.7	6.4	10.1	3.0	11.9	3.4	13.8	7.7	18.9	10.6	15.1	11.3
More than 21 glasses		18.2	5.2	9.7	2.6	7.7	2.5	9.9	2.7	14.3	4.9	12.5	3.1
Preferred beverage (N=14,482)													
Wine		3.2	39.9	16.7	60.4	26.1	68.1	34.3	80.7	39.4	82.6	46.3	85.3
Beer		72.3	14.4	62.9	10.9	50.7	8.4	46.9	5.3	40.6	3.9	25.8	2.3
Strong drinks		11.5	27.4	7.1	16.9	7.1	12.7	6.8	5.0	8.2	5.1	12.7	4.5
No preference		13.1	18.3	13.3	11.8	16.2	10.8	12.1	9.0	11.8	8.4	15.2	7.9
Urges to drink alcohol													
Social situations (N=15,115)	No	17.4	20.7	10.7	20.9	16.5	25.4	18.1	29.3	20.3	32.0	32.2	35.9
	Mild	53.3	59.3	55.1	57.5	57.0	57.8	63.4	58.4	66.4	59.1	60.4	58.3
	Strong	29.3	20.1	34.2	21.6	26.5	16.8	18.5	12.3	13.2	8.9	7.5	5.8
At dinner (N=15,038)	No	70.6	77.5	60.0	65.5	61.7	66.3	61.8	63.2	53.9	54.8	47.7	45.6
	Mild	28.3	21.1	36.6	30.6	34.1	29.2	33.6	32.2	40.3	40.6	46.2	51.0
	Strong	1.0	1.5	3.4	3.9	4.2	4.5	4.6	4.6	5.8	4.6	6.1	3.3
After work (N=14,892)	No	66.3	90.8	77.1	89.3	81.0	90.5	81.3	87.1	72.8	80.9	78.0	82.2
	Mild	26.8	8.4	20.1	9.0	16.7	7.6	15.3	10.1	22.8	15.6	18.5	16.4
	Strong	6.9	.8	2.8	1.7	2.3	1.9	3.5	2.9	4.4	3.5	3.5	1.4

		Age 18-25		Age 25-35		Age 35-45		Age 45-55		Age 55-65		Age 65 or older	
		Men *N=1,235* %	*Women* *N=2,499* %	*Men* *N=839* %	*Women* *N=1,769* %	*Men* *N= 995* %	*Women* *N= 1,891* %	*Men* *N=1,192* %	*Women* *N= 2,265* %	*Men* *N= 1,255* %	*Women* *N= 1,507* %	*Men* *N= 536* %	*Women* *N= 604* %
Relaxing (N=15,056)	No	36.4	52.8	40.2	52.7	40.5	51.7	29.5	41.8	25.9	40.6	36.8	38.1
	Mild	51.9	42.2	51.6	43.1	48.9	42.5	61.9	52.3	66.7	53.6	59.5	58.6
	Strong	11.7	4.9	8.2	4.2	10.6	5.8	8.6	5.8	7.3	5.8	3.7	3.3
Concentrating (N=14,735)	No	97.0	98.5	98.1	99.0	97.6	99.1	97.5	99.0	96.2	96.5	93.5	96.3
	Mild	2.8	1.5	1.8	.9	2.2	.8	2.3	.9	3.5	3.3	6.3	3.7
	Strong	.2	0	.1	.1	.2	.1	.2	.1	.3	.2	.2	0
Under stress (N=14,775)	No	79.9	84.1	80.3	83.9	82.1	82.6	78.2	77.6	72.9	73.0	75.1	73.4
	Mild	16.2	13.3	16.1	12.8	15.4	14.2	17.8	18.1	21.8	21.3	19.9	21.2
	Strong	4.0	2.6	3.6	3.3	2.5	3.2	4.0	4.3	5.3	5.6	5.0	5.4
No. of intoxications (N=9,056)													
Once or twice		25.8	36.7	13.4	30.1	18.4	36.7	22.3	44.1	26.2	50.7	36.3	55.3
3-5 times		26.5	32.3	20.8	30.5	21.3	34.4	33.3	36.0	32.8	29.7	31.8	28.5
6-10 times		18.1	15.5	23.2	17.3	19.5	16.4	23.6	13.1	22.3	12.3	18.0	10.6
11-25 times		14.2	8.6	14.2	12.4	16.0	7.6	10.5	3.9	10.4	4.5	9.0	4.9
More than 25 times		15.4	6.9	28.4	9.7	24.9	4.8	10.2	2.9	8.3	2.8	4.8	.8
Alcohol abuse disorder symptoms (N=15,288)		36.6	21.5	42.5	19.4	33.7	18.0	34.6	23.2	37.4	24.0	28.0	16.2
Hazardous drinking (N=15,516)		29.8	13.4	23.3	7.7	18.6	6.7	16.7	10.3	19.3	9.9	12.7	5.5
Age at alcohol initiation		14.4	14.5	15.1	15.4	15.6	16.4	15.4	16.5	16.2	17.8	18.1	20.4
(N=15,202)		(1.9)	(1.7)	(2.3)	(2.4)	(2.6)	(2.8)	(2.3)	(3.0)	(2.7)	(4.5)	(4.2)	(6.8)
Age at onset regular drinking		16.7	17.0	18.3	19.4	19.3	22.4	20.6	25.6	22.7	29.5	27.8	33.3
(N=8,638)		(1.4)	(1.6)	(2.9)	(4.5)	(4.1)	(6.1)	(5.9)	(9.0)	(8.4)	(11.1)	(12.0)	(12.7)
Age at first intoxication		16.6	16.7	17.6	18.8	18.3	20.8	18.7	22.2	20.6	25.4	24.3	32.2
(N=10,253)		(1.5)	(1.5)	(2.7)	(3.3)	(3.1)	(5.2)	(3.7)	(7.0)	(5.3)	(8.5)	(8.3)	(11.0)
