# Supplementary material for: Increases in alcohol consumption in women and elderly groups: evidence from an epidemiological study
Source: BMC Public Health. 2013 Mar 8;13:207. doi: 10.1186/1471-2458-13-207 (PMC3720280; doi:10.1186/1471-2458-13-207)
Supplement: Additional file 2: Table A2 — Cross-validation of regression (β) of alcohol use on demographic and lifestyle variables. [file 1471-2458-13-207-S2.pdf]

Table A2 Cross-validation of regression (β) of alcohol use on demographic and lifestyle variables

	age	*sex*	*age*sex*	*educ. attainm.*	*student*	*wrk stress*	*fin. stress*	*urban.*	*relig*	*smoking initiation*	*cannabis initiation*	*bmi*	R^2
Alcohol initiation (N=3,118)													
A few times to try	-.20 (.43)	**.51 (.20)**	.00 (.34)	.33 (.17)	.31 (.20)	.03 (.21)	-.08 (.20)	-.02 (.17)	-.21 (.16)	.64 (.17)	.19 (.32)	.01 (.17)	
Yes	-.11 (.14)	-.17 (.07)	.03 (.12)	.29 (.07)	.17 (.08)	.05 (.09)	-.06 (.09)	.10 (.07)	-.21 (.07)	.70 (.08)	.33 (.13)	-.09 (.07)	
Alcohol frequency (N=3,088)	.30 (.03)	-.20 (.02)	.01 (.03)	.13 (.02)	.10 (.02)	.02 (.02)	-.02 (.02)	-.01 (.02)	-.05 (.02)	.18 (.02)	.04 (.02)	-.08 (.02)	.18
Alcohol quantity (N=2,592)	.12 (.03)	-.26 (.02)	.04 (.03)	-.03 (.02)	.11 (.02)	.00 (.02)	.01 (.02)	-.04 (.02)	-.08 (.02)	.20 (.02)	.09 (.02)	-.03 (.02)	.16
Preferred beverage (N=2,820)													
Wine	.63 (.11)	.71 (.05)	-.06 (.11)	.36 (.06)	-.07 (.08)	.14 (.09)	-.08 (.09)	-.07 (.07)	.02 (.07)	.14 (.07)	-.08 (.07)	-.09 (.07)	
Beer	-.31 (.13)	-.87 (.04)	.19 (.14)	.09 (.09)	.02 (.09)	.15 (.11)	-.03 (.11)	-.28 (.07)	-.05 (.08)	.26 (.08)	.08 (.09)	.08 (.09)	
Strong liquor	.01 (.28)	.26 (.17)	-.93 (.20)	-.17 (.15)	-.11 (.16)	.38 (.18)	-.19 (.18)	-.18 (.15)	-.03 (.15)	.25 (.15)	-.38 (.15)	.24 (.15)	
Urge to drink alcohol													
Social situations (N=2,951)	-.17 (.03)	-.12 (.02)	**.08 (.03)**	.15 (.02)	.08 (.02)	.01 (.02)	.04 (.02)	.02 (.02)	-.02 (.02)	.11 (.02)	.14 (.02)	-.01 (.02)	.12
At dinner (N=2,934)	.16 (.04)	-.04 (.02)	.09 (.03)	.18 (.02)	.05 (.03)	-.04 (.03)	.02 (.03)	.09 (.02)	.02 (.02)	.01 (.02)	.08 (.03)	-.02 (.02)	.09
After work (N=2,921)	-.03 (.04)	-.25 (.03)	.13 (.04)	.03 (.03)	.03 (.03)	.02 (.04)	.03 (.03)	.02 (.03)	-.04 (.03)	.11 (.03)	**.12 (.03)**	-.02 (.03)	.12
When relaxing (N=2,932)	.01 (.03)	-.16 (.02)	.06 (.03)	-.01 (.02)	.02 (.02)	.05 (.02)	-.01 (.02)	**-.06 (.02)**	.02 (.02)	.11 (.02)	**.07 (.02)**	.01 (.02)	.06
Concentrating (N=2,890)	.24 (.12)	-.22 (.07)	-.15 (.11)	-.01 (.07)	.13 (.09)	-.11 (.09)	.14 (.08)	.03 (.07)	-.01 (.07)	.19 (.09)	-.02 (.07)	-.02 (.08)	.13
Under stress (N=2,896)	.19 (.04)	-.04 (.03)	-.04 (.04)	.04 (.03)	.03 (.03)	**.08 (.03)**	.04 (.03)	-.03 (.03)	-.05 (.03)	.14 (.03)	.11 (.03)	-.03 (.03)	.08
No. of intoxications (N=1,815)	-.07 (.03)	-.25 (.02)	-.05 (.03)	.07 (.02)	-.04 (.03)	-.08 (.03)	.09 (.03)	.01 (.02)	-.07 (.02)	.11 (.02)	.23 (.02)	.00 (.02)	.17
AAD symptoms (N=2,986)	.04 (.04)	-.19 (.02)	.03 (.04)	.10 (.02)	.07 (.03)	.06 (.03)	.02 (.03)	.00 (.02)	-.05 (.02)	.19 (.03)	.15 (.02)	-.02 (.03)	.14
Hazard. drinking (N=3,001)	-.09 (.04)	-.22 (.03)	.05 (.05)	.03 (.03)	.08 (.03)	.04 (.03)	.07 (.03)	-.02 (.03)	-.06 (.03)	.22 (.03)	.11 (.03)	-.03 (.03)	.15
Age alc. initiation (N=2,948)	.23 (.03)	.10 (.02)	.15 (.03)	-.06 (.02)	-.01 (.02)	-.04 (.02)	.01 (.02)	.05 (.02)	-.01 (.02)	-.13 (.02)	-.13 (.02)	-.03 (.02)	.18
Age onset reg. drink. (N=1,680)	.31 (.03)	.18 (.02)	.23 (.03)	.01 (.02)	.03 (.01)	-.09 (.02)	**.07 (.03)**	.05 (.02)	.03 (.02)	-.02 (.02)	-.07 (.02)	-.04 (.02)	.29
Age first intox. (N=2,008)	.32 (.03)	.17 (.02)	.18 (.03)	-.02 (.02)	.03 (.01)	-.01 (.02)	-.03 (.02)	.05 (.02)	.01 (.02)	-.08 (.02)	-.11 (.02)	-.04 (.02)	.24

Note. β (beta): standardized regression coefficients, with standard errors in parentheses. Betas in black font were significant at α=.01. Betas in grey were not significant (at α=.01). Betas in bold font were significant (at α=.01) in the validation sample but not observed in the main sample.
